# Inflaming the Brain with Iron

**DOI:** 10.3390/antiox10010061

**Published:** 2021-01-06

**Authors:** Pamela J. Urrutia, Daniel A. Bórquez, Marco Tulio Núñez

**Affiliations:** 1 Department of Biology, Faculty of Sciences, Universidad de Chile, 7800024 Santiago, Chile; pamela.urrutia.v@gmail.com; 2 Center for Biomedical Research, Faculty of Medicine, Universidad Diego Portales, 8370007 Santiago, Chile; daniel.borquez@udp.cl

**Keywords:** neuroinflammation, iron, Alzheimer’s disease, Parkinson’s disease, hepcidin, nitric oxide, iron regulatory protein 1, oxidative stress

## Abstract

Iron accumulation and neuroinflammation are pathological conditions found in several neurodegenerative diseases, including Alzheimer’s disease (AD) and Parkinson’s disease (PD). Iron and inflammation are intertwined in a bidirectional relationship, where iron modifies the inflammatory phenotype of microglia and infiltrating macrophages, and in turn, these cells secrete diffusible mediators that reshape neuronal iron homeostasis and regulate iron entry into the brain. Secreted inflammatory mediators include cytokines and reactive oxygen/nitrogen species (ROS/RNS), notably hepcidin and nitric oxide (·NO). Hepcidin is a small cationic peptide with a central role in regulating systemic iron homeostasis. Also present in the cerebrospinal fluid (CSF), hepcidin can reduce iron export from neurons and decreases iron entry through the blood–brain barrier (BBB) by binding to the iron exporter ferroportin 1 (Fpn1). Likewise, ·NO selectively converts cytosolic aconitase (c-aconitase) into the iron regulatory protein 1 (IRP1), which regulates cellular iron homeostasis through its binding to iron response elements (IRE) located in the mRNAs of iron-related proteins. Nitric oxide-activated IRP1 can impair cellular iron homeostasis during neuroinflammation, triggering iron accumulation, especially in the mitochondria, leading to neuronal death. In this review, we will summarize findings that connect neuroinflammation and iron accumulation, which support their causal association in the neurodegenerative processes observed in AD and PD.

## 1. Introduction

Brain iron overload in neurodegeneration-prone areas and in neuroinflammation has been broadly recognized as a pathological hallmark of neurodegenerative diseases, such Alzheimer’s disease (AD) and Parkinson’s disease (PD). Neuroinflammation refers to the inflammatory responses mediated by the innate immune system that take place in the central nervous system (CNS). Although it shares many features with peripheral inflammation, the coexistence of CNS specialized cell types, such as microglia, astrocytes, neurons, endothelial cells, and pericytes, confers unique characteristics to brain inflammation. Furthermore, the loss of integrity of the blood–brain barrier (BBB) found in neuroinflammatory conditions allows the infiltration of peripheral inflammatory cells, such as macrophages [1].

The initiation of the progressive inflammatory process in AD and PD can be traced to the neurodegeneration of noradrenergic (NA) neurons in the locus coeruleus (LC), which is the earliest and more severely affected area in PD (Braak stage 2), followed by dopaminergic neurons of substantia nigra (SN; Braak stage 3) and ultimately, by the neurodegeneration of hippocampal and cortical neurons (Braak stage 5) [2]. Interestingly, in the most recent Braak staging of AD, tau pathology is first observed in the LC, later spreading to the entorhinal cortex and finally to other neocortical regions [3,4,5], suggesting shared molecular mechanisms with PD [6].

The selective vulnerability of LC-NA neurons correlates with their higher production of reactive oxygen species (ROS) under physiological conditions, which is significantly potentiated by peripheral inflammation, resulting in mitochondrial damage. An elevated expression of neuronal NADPH oxidase (NOX), which catalyzes the production of the superoxide radical (O_2_^−^), plays an important role in the selective susceptibility of LC-NA neurons [7]. Interestingly, LC neurodegeneration can be triggered by an intraperitoneal lipopolysaccharide (LPS) injection [8], suggesting that a gut–brain axis may play a significative role in PD pathogenesis, probably associated with a “body-first” PD subtype [9].

In the brain, norepinephrine (NE) significantly contributes to the suppression of neuroinflammatory responses, by attenuating microglial surveillance and activation, reducing the secretion of proinflammatory factors, and decreasing phagocytic NOX2-mediated ·O_2_^−^ production [10,11,12,13,14]. Accordingly, the use of N-(2-chloroethyl)-N-ethyl-2-bromo-benzylamine (DSP-4), which is a selective NE toxin, potentiates neuroinflammation induced by amyloid β (Aβ)_1–42_ aggregates [15] or bacterial endotoxin lipopolysaccharide (LPS) [16,17] and promotes AD and PD pathogenesis in several animal models [16,18,19,20,21,22,23,24,25,26].

Microglia/macrophage activation can be followed during the progression of neurodegeneration by non-invasive techniques, such as positron emission tomography (PET), using radiotracers specifically designed for targeting the mitochondrial translocator protein 18-kDa (TSPO), which is a protein highly expressed in activated microglia/macrophages. Microglial/macrophage activation has been observed using PET in monkeys injected with the mitochondrial complex I inhibitor 1-methyl-4-phenyl-1,2,3,6-tetrahydropyridine (MPTP), which is a toxin that selectively kills dopaminergic neurons [27,28], and in rats expressing human A53T mutated α-synuclein in SN [29,30] or injected with the highly oxidable dopamine analog 6-hydroxydopamine (6-OHDA) [31,32]. Altered glial immune responses have also been observed in animal models of familial PD [33,34] and in transgenic mice expressing AD-associated mutant proteins [35,36]. An increase in TSPO binding has been consistently observed in studies with AD [37] and PD [38] patients. However, there are several concerns about potential artifacts in microglial TSPO PET imaging, including binding to multiple cell types, such as astrocytes and endothelial cells [39,40]; differential tracer affinity in TSPO Ala147Thr polymorphism carriers [41]; and other confounding factors [42,43]. Therefore, the conclusions of these studies should be interpreted with caution. Interestingly, a recent study on AD transgenic rats shows TSPO upregulation in astrocytes before microglia [44], urging the development of more specific tracers for studying the respective contributions of astrogliosis and microgliosis to the neurodegenerative process. Overall, the reported evidence points to a central role of neuroinflammation in the initiation and progression of neurodegenerative processes.

The activation of microglial cells triggers the release of diffusible mediators, including cytokines, ROS, and reactive nitrogen species (RNS). Remarkably, ROS/RNS generation is supported by two enzymatic systems: The NOX2 enzyme complex that synthesizes ·O_2_^−^, which, through its dismutation, generates hydrogen peroxide (H_2_O_2_), and the inducible form of nitric oxide synthase (iNOS), which generates ·NO. These enzymatic systems play a crucial role in AD- and PD-associated neurodegeneration, as revealed by the neuroprotection achieved by the pharmacological or genetic inhibition of NOX2 or iNOS reported in animal models of AD [45,46] and PD [47,48,49,50].

Clinical evidence from patients displaying chronic use of non-steroidal anti-inflammatory drugs (NSAID) shows a reduced risk for AD [51,52] and PD [53]. Based on these epidemiological observations and the beneficial effects of NSAID in AD animal models, several clinical trials have been conducted to assess their efficacy in AD and dementia. Unfortunately, these studies have shown no significant effects on the cognitive performance in AD patients, prompting improvement of the therapeutic window and the use of more selective inhibitors in future clinical trials (reviewed in [54]).

Recently, neuroinflammation has been associated with the alteration of iron homeostasis, and at the same time, iron dyshomeostasis has been shown to play a pivotal role in the neuroinflammatory phenotype. As a result, neuroinflammation and iron are entangled in a circuit that amplifies ROS production, leading to neuronal death. An analysis of postmortem tissue from PD patients shows significant elevations in the concentration of iron in the SN, where degenerating neuromelanin-bearing dopaminergic neurons reside [55,56]. Similarly, iron is concentrated in and around AD senile plaques [57,58], in Huntington’s disease basal ganglia [59], and in the spinal cord of sporadic amyotrophic lateral sclerosis patients [60]. Due to its paramagnetic property, iron’s content can be estimated in specific brain areas using magnetic resonance imaging (MRI), by measuring the R2* relaxation rate, phase changes in susceptibility-weighted imaging (SWI), or susceptivity values upon quantitative susceptibility mapping (QSM) [61] [62,63]. Neuromelanin-sensitive MRI has also been proposed as a diagnostic tool for PD [64]. Significant increases in iron levels are measured in vivo by iron-sensitive MRI, even in the early stages of AD and PD patients, showing a good correlation with the severity of their symptoms [63,65,66]. Patients with familial PD-associated mutations also display increased brain iron deposition by MRI, even in asymptomatic stages [67], suggesting that iron accumulation plays a role in the progression of the idiopathic and genetic forms of PD.

Iron overload is also associated with several animal models of AD and PD. Transgenic mice for Amyloid precursor protein/presenilin-1 (APP/PS1) [68,69,70,71] and 5xFAD [72] exhibit increased brain iron levels. Moreover, an injection of MPTP, rotenone, or 6-OHDA phenocopies many aspects of PD in rodents, including iron accumulation in the SN [73,74,75]. Supporting a causal role of iron accumulation in neurodegeneration, neonatal iron supplementation in mice triggers the progressive neurodegeneration of SN dopaminergic neurons, reduces striatal dopamine levels, and increases the responsiveness to MPTP insult [76]. Moreover, chronic oral administration of iron induces iron accumulation in specific brain regions, including the SN and caudate/putamen. Iron accumulation is associated with oxidative stress-related dopaminergic neuronal apoptosis in the SN and with motor and cognitive deficits [77]. Consequently, iron chelation prevents neuronal death in several animal models of AD and PD [78,79,80,81,82] and iron chelation has recently been introduced as a new therapeutic concept for the treatment of PD [83,84]. Nevertheless, the results on the use of iron chelation treatment demonstrate that it slows that disease progression [85]. Due to the multifactorial nature of the neurodegenerative process in PD, a single target treatment, such as the use of chelators, may not fully stop the neurodegenerative process. Accordingly, treatment with multifunctional compounds with an iron chelating capacity and aimed at reducing two or more of the pathological events associated with the progress of the disease (a “multi-target” approach) may be better suited for the treatment of PD [85,86]. 

Aging is the main risk factor for the development of sporadic forms of AD and PD, and both iron accumulation and neuroinflammation exhibit an age-synchronous increment in the brain. Iron levels and microglial and astrocytic numbers are positively correlated in aged mice basal ganglia [87] and iron-retentive microglia concurring with elevated iron levels and oxidative stress in aged non-human primates [88]. Interestingly, a genetic predisposition to neuroinflammation aggravates the striatal iron-related poor cognitive switching ability in aged humans [89], highlighting the intimate relationship between iron and neuroinflammation during aging (reviewed in [90]).

Correspondingly, in this review, we present a summary of the mechanisms that underlie the bidirectional relationship between iron and neuroinflammation and its relevance to AD and PD pathogenesis.

## 2. Iron Homeostasis in the CNS

Iron is an essential protein cofactor that performs a myriad of unique functions in the CNS, including ribosome assembly, DNA repair, mitochondrial energy production, metabolite catabolism, myelination, and neurotransmitter anabolism and catabolism [91]. In excess, however, iron is linked to cellular death, causing sustained cellular oxidative stress by the iron-mediated catalytic conversion of H_2_O_2_ and ·O_2_^−^ into toxic hydroxyl radicals as a result of Fenton and Haber–Weiss chemistry, respectively [92]. Accordingly, iron homeostasis must be tightly controlled.

Transferrin (Tf), which is a glycoprotein that possesses two high-affinity iron (III)-binding sites, is the primary iron transporter into the CNS and thus plays an essential role in cellular iron uptake. Following transferrin binding to its surface receptor, TfR1, the Tf-TfR1 complex is endocytosed through clathrin-dependent pathways into the early endosome, in which its low pH induces iron dissociation from Tf. The ferrireductase Steap2 reduces Fe^3+^ to Fe^2+^, which is transported into the cytoplasm by the divalent metal transporter-1 (DMT1). The apoTf/TfR1 complex returns to the plasma membrane, where the neutral pH induces its dissociation [93,94].

In the cytoplasm, iron is incorporated into the cytosolic labile iron pool (cLIP), which is distributed to three destinations: (i) To mitochondria, for the synthesis of iron-sulfur (Fe-S) clusters and heme prosthetic groups; (ii) to the cytoplasmic iron storage protein ferritin (Fn); or (iii) back to the extracellular fluid through the iron exporter, Fpn1. Ferritin is a multimeric protein assembled by 24 subunits of H and L monomers in a variable ratio, depending on the cellular type. The H subunit contains ferroxidase activity, while the L subunit is responsible for iron turnover at the ferroxidase site and iron nucleation within the Fn core [95].

Iron delivery to the brain is tightly regulated at the level of the BBB [94], composed of tight junction-adhered endothelial cells that safeguard the free access of molecules to the brain. Iron transport across the BBB is mediated by three mechanisms. Overall, the mechanism of iron transport across the BBB involves two transmembrane steps: Iron uptake at the luminal membrane of the brain capillary endothelial cells, followed by iron efflux into the brain interstitium at the abluminal membrane. The predominant mechanism involves the transcellular transport of iron through Tf endocytosis, DMT1-mediated transport from the endosome lumen into the cytoplasm, and Fpn1-mediated extrusion at the abluminal membrane [96,97,98]. A second mechanism involves Tf/TfR1 complex transcytosis across the endothelial cell and the release of Tf into the parenchyma at the abluminal membrane [99]. A third mechanism is dependent on Fn, which is present in blood serum and cerebrospinal fluid (CSF) [100,101,102]. Serum Fn is mainly composed of L subunits with one or two H subunits [95]. Both in vitro and in vivo studies have shown the transport of Fn across the BBB, utilizing different receptors [103,104,105]. The Scara5 receptor recognizes L-Fn [106], while H-Fn binds to TfR1 [107].

Iron released by brain vascular endothelial cells is quickly captured by nearby astrocytes, which play a critical role in regulating brain iron absorption at the abluminal side. Astrocytes do not express TfR1; however, DMT1 expression is highly polarized in astrocytes, in which DMT1 is mainly found in the end-foot processes associated with the BBB [108]. Therefore, iron released by the endothelial cells is probably taken up by nearby astrocytes through DMT1 and distributed to the brain parenchyma through Fpn1 [109]. The concentration of iron in the CSF ranges between 0.2 and 1.1 µM, whereas the concentration of Tf is about 0.24 µM [110,111]. Therefore, CSF iron levels often exceed the binding capacity of Tf [112] and iron is incorporated by neurons and glia from two sources: Transferrin-bound iron (TBI), through the Tf-TfR1 system, and non-transferrin bound iron (NTBI), through DMT1 or other iron transporters.

## 3. Role of Hepcidin in Neurodegeneration

Complex living organisms have developed sophisticated mechanisms to finely coordinate iron homeostasis and avoid iron overload. There is systemic iron regulation, mediated by hepcidin, and cellular iron regulation, through the iron regulatory element/iron regulatory protein (IRE/IRP) system. Both regulatory mechanisms are intertwined in a bidirectional relationship with inflammatory mediators, as detailed in the following text.

The peptide hormone hepcidin, mainly secreted into the bloodstream by hepatocytes, is the principal regulator of systemic iron homeostasis. Hepcidin controls dietary iron absorption, iron recycling by macrophages, and iron release from hepatic stores through the regulation of iron transporter levels, generating a decrease of iron plasma levels [113]. In enterocytes, hepcidin induces the internalization and proteasomal degradation of apical-side DMT1, limiting early dietary iron absorption [114,115]. In comparison, in reticulo-endothelial cells (splenic macrophages and Kupffer cells in the liver) and hepatocytes, hepcidin binds to the Fpn1 C-terminal domain in an iron-dependent way, inducing its endocytosis and subsequent Fpn1 lysosomal degradation, enhancing iron sequestration [113,116,117].

Hepcidin expression is regulated by plasma iron levels, inflammation, and erythropoiesis. Regulation by iron plasma levels involves multiple pathways by which hepatocytes sense the circulating iron status. One pathway involves the secretion of iron-induced bone morphogenic protein (BMP) by liver sinusoidal endothelial cells [118]. BMP6 and BMP2 bind to the BMP receptor, triggering the phosphorylation and activation of SMAD1/5/8, which, complexed with SMAD4, translocates to the nucleus to induce hepcidin transcription [119,120]. Hepatocytes also sense plasma iron levels through the interaction of HFE with TfR1 and TfR2. Under low iron conditions, HFE binds to TfR1. Under high iron conditions, the binding of Tf-Fe to TfR1 displaces HFE that then binds to TfR2. The HFE/TfR2 complex interacts with hemojuvelin (HJV), potentiating the BMP signaling pathway and hepcidin transcription [121,122]. The inflammatory cytokines IL6, IL1β, and IL22 induce hepcidin expression in hepatocytes through activation of the STAT3 signaling pathway [123,124,125]. The BMP/SMAD pathway is also involved in the regulation of hepcidin transcription downstream of inflammatory stimuli [126].

Since iron is required for hemoglobin synthesis, hepcidin expression is suppressed during erythropoiesis. The main erythroid regulator of hepcidin is erythroferrone, which is synthesized and secreted by developing erythroid cells [127], reviewed in [128]. Erythroferrone acts on hepatocytes, suppressing the production of hepcidin through a mechanism that involves targeting of the SMAD1/5 signaling pathway [129].

Hepcidin expression has also been described in the CNS. Hepcidin mRNA has been detected in several brain regions, including the cortex, hippocampus, amygdala, thalamus, hypothalamus, olfactory bulb, mesencephalon, cerebellum, pons, and spinal cord [130,131,132]. In the human brain, hepcidin has been detected in endosomal structures in reactive astrocytes and epithelial cells of the choroid plexus, colocalizing with Fpn1 [133]. Interestingly, during aging, both hepcidin mRNA and protein levels increase in the cerebral cortex, hippocampus, striatum, and SN [131,134]. The hepcidin peptide is also localized in the endothelium of blood vessels, choroid plexus, and pericytes [135], suggesting that brain hepcidin originates from both in situ production and systemic production [135,136]. Cell culture experiments showed that hepcidin is produced by microglia and astrocytes, as well as by pericytes [137,138].

Resembling the regulation of dietary iron absorption in the duodenum, hepcidin acts at the BBB, reducing iron entry into the brain. Hepcidin knockout (KO) mice show strongly increased Fpn1 immunoreactivity at the abluminal side of vascular endothelial cells [139], suggesting a reduction in Fpn1 turnover. At the BBB, hepcidin is secreted in a synaptic-like manner by astrocytes and only stimulates Fpn1 internalization and degradation in vascular endothelial cells in close proximity to astrocytes’ end-feet, reducing iron export [104,140,141].

Hepcidin expression in the brain is regulated by inflammatory stimuli; for example, LPS and turpentine oil induce hepcidin expression in the cortex, hippocampus, and striatum [142,143,144]. Peripheral LPS administration also increases hepcidin mRNA and protein levels in the cerebral cortex, SN [145], and choroid plexus [146]. However, more studies are required to determine the contribution of peripherical inflammation upon brain iron homeostasis. Interestingly, the hepcidin expression in astrocytes is mainly dependent on the IL6-STAT3 pathway, since LPS treatment of IL6 null-derived primary cultures fails to induce an increase of hepcidin mRNA levels, in contrast with a robust induction in wild-type cultured cells [147]. The proposed mechanism involves LPS-mediated IL6 secretion from microglia, and the subsequent IL6-triggered hepcidin production in astrocytes by means of a STAT3-mediated pathway [148]. In astrocytes, hepcidin knockdown reduces the neuronal iron accumulation, oxidative stress, and apoptosis generated by an LPS intraventricular injection [148], suggesting a deleterious role of hepcidin in neuroinflammation.

Understanding the function of hepcidin in the CNS is an ongoing process. Hepcidin can prevent iron accumulation in the brain, by inhibiting TfR1, DMT1, and Fpn1 expression on microvascular endothelial cells and thus reducing TBI and NTBI uptake [149]. However, several studies show conflicting results regarding the role of hepcidin in several neuronal pathologies. These discrepancies can partially be explained by the use of isolated cell cultures, which do not take into account the interaction between neurons and glial cells observed in the intact brain. Accordingly, hepcidin loss-of-function protects N27 rat dopaminergic cells from 6-OHDA-induced apoptosis, decreasing the intracellular iron content and oxidative stress [150]. In contrast, in vivo hepcidin overexpression in astrocytes prevents the increase in brain iron levels and oxidative stress in a systemic iron overload rat model [151] and reduces dopamine neuronal loss and limits iron accumulation in the SN in rotenone and 6-OHDA animal models of PD. Remarkably, hepcidin overexpression also promotes α-synuclein clearance through autophagy, reduces mitochondrial dysfunction, and improves motor deficits [152,153].

A protective role for hepcidin has also been reported in AD models. Hepcidin pre-treatment reduces the secretion of inflammatory cytokines induced by the Aβ peptide and decreases the toxicity of astrocytes and microglia conditioned media in hippocampal neurons [154]. Moreover, hepcidin pretreatment reduces both the oxidative damage and the glial activation in the hippocampus displayed by animals after an intraventricular injection of Aβ [154]. Accordingly, in APP/PS1 transgenic mice, hepcidin overexpression by astrocytes reduces iron entry into the brain and diminishes iron accumulation in neurons, which results in decreased neuronal death in the cortex and hippocampus [155]. Interestingly, one study shows that hepcidin and Fpn1 are reduced in post-mortem tissue from AD patients [156], suggesting a key role of hepcidin in the development of this disease.

Overall, these findings suggest that hepcidin secretion by astrocytes exerts a spatially restricted action on endothelial cells, reducing iron entry into the brain and providing neuroprotection. On the other hand, under neuroinflammation, unleashed hepcidin expression triggered by IL6 can generate iron accumulation in neurons, promoting neurodegeneration.

## 4. Neuroinflammation Modulates the IRE/IRP System in Neurodegeneration

Changes in the cell iron status (iron overload or depletion) lead to compensating translational changes in the levels of iron homeostasis-related proteins through the iron regulatory element/iron regulatory protein (IRE/IRP) system. Inflammatory mediators (especially ·NO) can target the IRE/IRP system, completely reshaping iron homeostasis in neurons and glial cells and amplifying the neurotoxic effects of unresolved neuroinflammation. Two IRP isoforms, known as IRP1 and IRP2, modulate the expression of proteins by binding to conserved stem-loop structures, named IREs, in the untranslated regions (UTRs) of their mRNAs. The regulatory outcome depends on the position and context of the IRE in the mRNA sequence: IRP binding to the 5′ UTR IRE region represses translation, whereas IRP binding to the 3′ UTR IRE region indirectly stimulates translation through the suppression of mRNA degradation [157]. In iron-deficient cells, IRPs selectively bind IRE at the 5′ UTR region of the mRNA coding for Fn and Fpn1 and to 3′ UTR of the mRNA coding for TfR1 and DMT1, promoting iron uptake. In conditions of iron excess, IRP2 is degraded and the IRP1 apoprotein binds to a [4Fe-4S] cluster to convert it into cytosolic (c)-aconitase, suppressing its RNA-binding activity [158,159]. Diminished IRP binding to the IREs promotes Fn and Fpn1 synthesis, whereas the TfR1 and DMT1 mRNAs are degraded by nucleases.

As mentioned above, IRP1 is a bifunctional cytoplasmic protein that transits reversibly between two conformations: An active RNA-binding protein (properly IRP1) and a [4Fe-4S] cluster-bearing protein, inactive for RNA binding that functions as a c-aconitase. The c-aconitase has an exclusively dedicated maturation system by the cytosolic Fe-S cluster protein assembly (CIA) machinery, where the heterotrimeric complex (CIA2A)_2_CIAO1 transfers one [4Fe-4S] cluster to IRP1, generating the active c-aconitase [160,161]. The CIA system depends on the mitochondrial Fe-S cluster assembly machinery (ISC). Therefore, IRP1 accumulates under iron deficiency conditions, when the ISC assembly machinery is impaired, acting as a sensor for the availability of mitochondrial iron and ensuring an adequate iron supply to this organelle [162,163].

Increased IRP1 IRE-binding activity has been observed in cells deficient in glutaredoxin 2 (GLRX2) [164], glutaredoxin 5 (GLRX5) [165], sideroflexin 4 (SFXN4) [166], or frataxin (FXN) [162], all of which are essential proteins for Fe-S cluster assembly. As IRP1 directs iron flux preferentially to the mitochondria, its unphysiological activation generates mitochondrial iron overload, and a deficiency in the availability of iron in the cytoplasm, which further potentiates iron entry into the cell through increases in TfR1 and diminished Fn levels [164,165,166]. 

Recently, an IRP1-dependent mitophagy activation mechanism has been described, suggesting that IRP1 could control mitochondrial iron recycling, analogous to the recycling of amino acids through macroautophagy. Mitophagy activation involves IRP1 binding to the 5′ UTR IRE sequence on Bcl-xL mRNA, repressing its translation in cells under iron depletion or impaired Fe-S cluster biogenesis [167]. This mechanism is consistent with early observations showing that deferiprone, which is an iron chelator, specifically activates mitophagy rather than macroautophagy [168].

**Figure 1:**
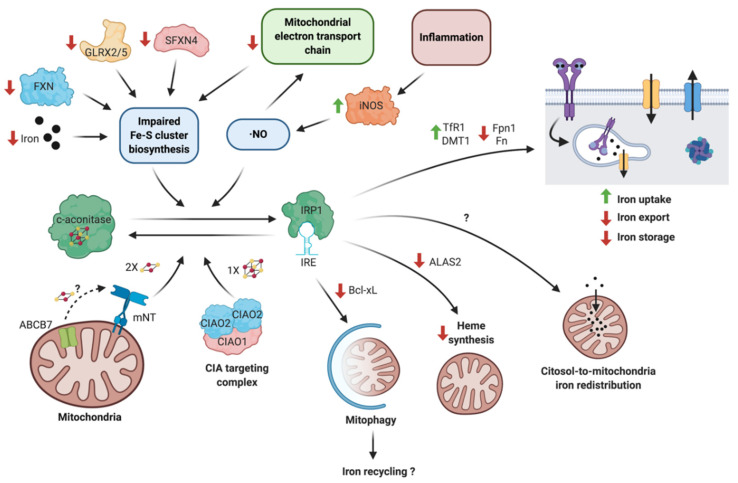
Regulatory mechanisms and functions of iron regulatory protein 1 (IRP1). The bifunctional protein c-aconitase/IRP1 is regulated by several mechanisms. C-aconitase is converted to IRP1 through two main processes: (i) Impaired Fe-S cluster biosynthesis, as a result of decreased mitochondrial iron availability, defects in the Fe-S cluster assembly (ISC) machinery, or bioenergetic failure, or (ii), through downstream inflammation by ·NO-mediated Fe-S cluster disruption. Conversely, IRP1 is turned back into c-aconitase by mNT under oxidative stress or by a specialized branch of the CIA targeting complex. The binding of IRP1 to iron response elements (IRE) in specific mRNA targets regulates mitophagy, heme synthesis, iron redistribution to mitochondria, and iron uptake and storage. FXN: frataxin; GLRX2/5; Glutaredoxin-2/5; SFXN4: Sideroflexin 4; CIA: cytoplasmic iron-sulfur assembly; and mNT: mitoNEET. Created with BioRender.com.

Through the regulation of erythroid-specific aminolevulinate synthase 2 (ALAS2), IRP1 also balances iron availability and its utilization by mitochondria. ALAS2 catalyzes the first step of heme biosynthesis and is negatively regulated by IRP1 binding to the 5′ IRE sequence in ALAS2 mRNA. Under mitochondrial iron-deficient conditions induced by mitoferrin-1 deficiency, IRP1 activation and subsequent ALAS2 translation inhibition prevent the accumulation of protoporphyrin, which are the precursors of the heme group [169]. Heme binding also inhibits IRPs, since the heme concentration is expected to increase under mitochondrial iron sufficiency conditions. Heme binding decreases IRP activity by steric competence with IREs or by oxidatively-mediated degradation [170,171]. These IRP1 regulatory mechanisms and associated effectors are summarized in Figure 1.

Both IRP1 and IRP2 mostly share their target mRNAs, but IRP2 is activated through a different mechanism when compared to IRP1. Under iron sufficient conditions, IRP2 is constitutively degraded by the proteasome. The E3 ubiquitin ligase FBXL5 controls IRP2 polyubiquitination. In turn, FBXL5 is also regulated by the ubiquitin-proteasome system. The FBXL5 ligase has an N-terminal hemerythrin-like domain with a di-iron center, which allows its correct folding and provides protection against degradation [172,173,174]. Under iron deficiency conditions, the N-terminal domain partially unfolds and is polyubiquitated by the HERC2 ubiquitin ligase [175]; FBXL5 also has a redox-sensitive [2Fe-2S] cluster in the C-terminal substrate recognition domain, which, upon oxidation, promotes IRP2 binding in an oxygen-dependent manner [176]. The CIA targeting complexes CIAO1, CIAO2B, and MMS19 exhibit an oxygen-dependent interaction with the C-terminal of FBXL5, potentiating IRP2 degradation [177]. These findings strongly suggest that these complexes could transfer the [2Fe-2S] cluster to FBXL5.

IRP1 KO mice show an apparently normal phenotype with tissue-specific iron dysregulation in brown fat and kidneys. Increased HIF2α translation in the kidney of IRP1 KO juvenile animals leads to increased erythropoietin expression, splenomegaly, and polycythemia, although this phenotype is normalized in adult animals [178,179]. The HIF2α mRNA contains a 5′ IRE sequence preferentially recognized by IRP1, which would explain the selective effect on the IRP1-null background. In contrast, IRP2 KO mice exhibit the misregulation of iron homeostasis in several tissues, including the brain, duodenum, and bone marrow, which IRP1 fails to compensate for [180,181]. Accordingly, FBXL5-null mice die during embryonic development because of iron overload and oxidative stress, although the deletion of IRP2, but not IRP1, restores the viability [182,183].

In tissues, IRP1 is mainly found as c-aconitase and has a limited contribution to the control of iron homeostasis under physiological conditions, not significantly responding to iron starvation [181]. Interestingly, IRP1 is a poor iron sensor at low (tissular) oxygen tension, but it becomes relevant at 21% oxygen (cell culture conditions), suggesting that oxygen-derived reactive species are key to IRP1 activation [184]. For example, tempol, which is a nitroxide radical, can activate the large latent reservoir of IRE-binding activity in the form of c-aconitase, restoring iron homeostasis in an IRP2-null background [185]. 

In summary, IRP1 acts as a sensor for mitochondrial iron deficiency, activating mitophagy to recycle the iron contained in this organelle, and stimulating iron uptake to restore mitochondrial iron homeostasis, whereas IRP2 outcompetes IRP1 in the regulation of cellular iron homeostasis in physiological conditions. Inflammatory mediators such as ROS/RNS can trigger decomposition of the [4Fe-4S] cluster of c-aconitase, activating IRP1, even under iron sufficiency conditions. This paradoxical activation plays an important role in neurodegenerative processes. An intricate scenario is generated by inflammatory cell activation, which produces a diverse repertoire of ROS/RNS. In addition to ·O_2_^−^, H_2_O_2_, and ·NO produced by enzymatic systems, the highly reactive peroxynitrite and hydroxyl radical generated non-enzymatically also form part of this repertoire. Due to their differential reactivity and diffusibility, each ROS/RNS affects the Fe-S cluster of c-aconitase in a different way.

The superoxide anion attacks the c-aconitase, leading to Fe-S cluster loss and IRP1 activation. Cytosolic superoxide dismutase (SOD1) (but not mitochondrial SOD2) confers selective protection to c-aconitase, suggesting that ·O_2_^−^ action is limited to its compartment of origin [186]. Moreover, the ·O_2_^−^-dependent intracellular oxidative stress observed in SOD1-null mice drastically reduces IRP1 protein levels [187,188]. This adaptive regulation can be facilitated by FBXL5-mediated IRP1 degradation, thus preventing excessive IRE-binding activity [189]. Hence, under inflammatory conditions, ·O_2_^−^ is generated extracellularly and does not activate IRP1 [190].

In vitro, H_2_O_2_ converts purified c-aconitase into the [3Fe-4S] form, losing its aconitase activity, without eliciting IRE-binding activity [191]. Accordingly, only extracellular H_2_O_2_ (and not intracellular H_2_O_2_) triggers the conversion of c-aconitase into active IRP1 [190,192], indicating that H_2_O_2_ acts indirectly. Experiments performed on permeabilized cells show that the conversion of c-aconitase to IRP1 triggered by H_2_O_2_ requires membrane-associated components [193], strongly suggesting the participation of a signaling-mediated event [194]. The consequences of extracellular H_2_O_2_ treatment on human neuroblastoma cells, including Fpn1 degradation, IRP1-mediated H-Fn protein level reduction, and increased cLIP have been described [195].

The most important direct activator of the IRE-binding activity of IRP1 is ·NO, acting as the main transducer of ·NO on iron metabolism. Nitric oxide triggers the conversion of c-aconitase to IRP1 through a disassembly of its [4Fe-4S] cluster [196,197,198], and therefore, it has no effect on IRP2 activity [199,200]. However, ·NO only prompts the priming of apo-IRP1 for IRE binding; thioredoxin-mediated reduction of apo-IRP1 is needed for full RNA-binding activity [201]. Nitric oxide-mediated IRP1 activation increases TfR1 levels. Nitric oxide also activates H-Fn, L-Fn, and Fpn1 transcription, but parallel IRP1 activation represses its translation, resulting in largely preserved protein levels. Nitric oxide also induces Fe-S cluster disruption of mitochondrial (m)-aconitase, and IRP1 activation is essential for [4Fe-4S] cluster reconstitution, reinforcing its important role in mitochondrial iron sufficiency [200]. Finally, peroxynitrite disrupts the Fe-S cluster on c-aconitase and additionally induces tyrosine nitration of IRP1, inhibiting both aconitase and IRE-binding activity [196,197,202,203].

Upon oxidative disruption of the [4Fe-4S] cluster, IRP1 is quickly turned back into c-aconitase through a protein synthesis-independent mechanism [204]. This recycling pathway is mediated by mitoNEET (mNT), which is a dimeric [2Fe-2S] cluster-bearing protein located in the outer mitochondrial membrane. The mNT Fe-S cluster is resistant to H_2_O_2_ and ·NO-mediated decomposition and each monomer successively transfers its cluster to reconstitute c-aconitase [205]. Interestingly, only the oxidized state of the mNT Fe-S cluster is competent for transfer [206]. Remarkably, mNT KO mice exhibit iron accumulation, mitochondrial dysfunction, decreased striatal tyrosine hydroxylase (TH), and dopamine levels and motor deficits, representing many of the characteristics of early neurodegeneration in PD [207], underlining the importance of this pathway in avoiding the hyper activation of IRP1 under oxidative stress.

The paradoxical activation of IRP1 despite elevated iron levels has been observed under inflammatory conditions and/or under unrestricted ROS/RNS production, leading to a positive feedback loop that generates iron overload and cell death [208,209]. For example, rotenone boosts ROS production through mitochondrial complex I inhibition, increases TfR1 and DMT1 and decreases Fpn1 protein levels, and enlarges the cLIP in an IRP1-dependent manner. Accordingly, IRP1 silencing abolishes the rotenone-induced iron uptake increase and reduces complex I inhibition-triggered neuronal death [210].

Inflammatory cytokines can enhance iron accumulation by regulating IRP1 activity through ·NO-dependent and -independent mechanisms [211]. In rat hepatoma cells, treatment with interferon (IFN)-γ/tumor necrosis factor (TNF)-α/LPS triggers ·NO-mediated IRP1 activation without changes in IRP2, accompanied by the translational repression of Fn expression [199]. Similarly, in primary cultured hippocampal neurons, the pro-inflammatory cytokines TNFα and IL6 and the Toll-like receptor (TLR)-4 agonist LPS directly upregulate both the mRNA and protein levels of DMT1 and induce a transient decrease in Fpn1 protein levels, generating an increment of the iron content in neurons [137,212], which could be associated with IRP1 activation. Moreover, in primary cultures of ventral mesencephalic neurons, the pro-inflammatory cytokines IL1β and TNFα also promote iron influx and decrease iron efflux. Consistently, TfR1 and DMT1 (+IRE) are upregulated and Fpn1 is downregulated. These changes are mediated by the ·NO- and ROS-mediated activation of IRP1, downstream of pro-inflammatory cytokines [213].

In vivo evidence also supports the role of paradoxical IRP1 activation in neurodegenerative diseases. Early findings showed that sustained IRP1 activity in PD can repress Fn translation, despite increased iron levels [214]. Similarly, IRP1 forms a more stable complex with IREs in AD brains, which could explain the absence of Fn upregulation [215]. The unexpected finding of an IRE sequence selectively recognized by IRP1 in the 5´UTR of APP mRNA generated a link between iron accumulation and Aβ deposition [216,217]. Additionally, IL1β stimulates IRP1 binding to APP mRNA IRE, suggesting that inflammatory stimuli can decrease APP translation [216]. Two putative mechanisms have been proposed to explain the link between APP and iron homeostasis: APP interaction with Fpn1, in order to provide the necessary ferroxidase activity for the oxidation and transfer of the exported iron to Tf [218], and APP-mediated membrane Fpn1 stabilization [219,220,221]. Moreover, recent findings strongly suggest that ·NO-mediated IRP1 activation diminishes APP levels and iron export in PD, promoting iron deposition [222]. In summary, ROS/RNS produced during inflammatory oxidative bursts can activate IRP1, promoting iron overload and neuronal death. Likewise, this mechanism has consequences on the inflammatory cells themselves, as addressed below.

## 5. Iron and Microglia/Macrophage M1/M2 Polarization

Macrophages and microglia play crucial roles in homeostatic and immune defense in the CNS. Upon infection or tissue injury, resident microglia are activated and peripheral macrophages are recruited to the CNS to eliminate pathogens or damaged cells. Additionally, microglia and macrophages have an anti-inflammatory or “resolving” function associated with tissue repair. Although tissular macrophages/microglia possess a broad spectrum of phenotypes, a simple bi-state model of inflammatory/classical (M1)- and resolution/alternative (M2)-activated macrophages/microglia has been widely used [223]. As described later in the text, M1 and M2 macrophages/microglia exhibit different iron homeostasis settings and their own differentiation is influenced by this metal ion.

**Figure 2:**
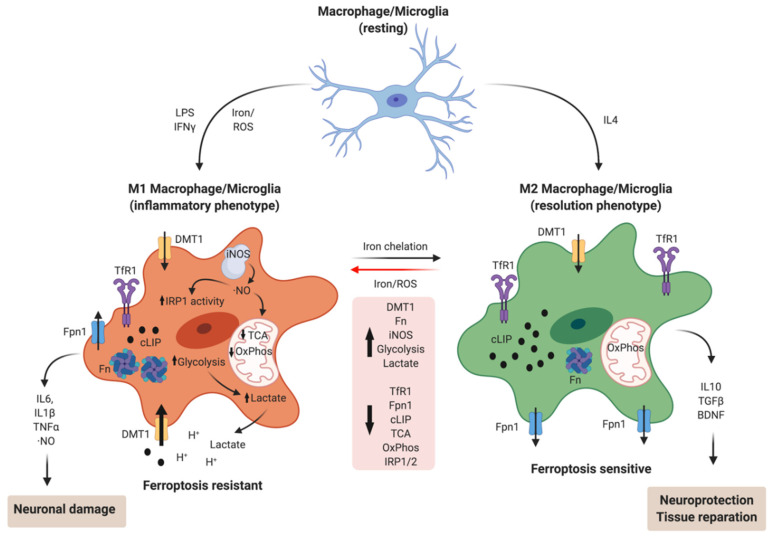
Iron homeostasis during macrophage/microglia M1/M2 polarization. Iron plays a central role in the balance between M1 inflammatory and M2 resolving phenotypes, stimulating M1 differentiation or converting the M2 phenotype into M1. In addition, ·NO generated by M1 macrophages/microglia reshapes cellular iron homeostasis, diminishing the cytosolic labile iron pool (cLIP) and reducing mitochondrial oxidative metabolism, thus conferring resistance to ferroptosis. Conversely, the M2 phenotype is ferroptosis-prone because of higher cLIP, energy dependence on oxidative phosphorylation, and the production of lipid oxidation products. Created with BioRender.com.

The M1 phenotype can be induced by LPS and IFNγ treatment and is characterized by increased iNOS expression and the secretion of inflammatory cytokines such as IL6, IL1β, and TNFα. On the other hand, M2 phenotype differentiation can be achieved by IL4 treatment and is characterized by increased arginase-1 levels and secretion of the brain-derived neurotrophic factor (BDNF), anti-inflammatory cytokines such as IL10, and several lipid mediators [223]. Interestingly, M1/M2 macrophages possess a completely opposite phenotype of iron handling (Figure 2). While M1 macrophages have lower IRP binding activity, low cLIP, lower levels of TfR1 and Fpn1, and higher levels of H-Fn, M2 macrophages have higher IRP binding activity, a larger cLIP, higher TfR1 and Fpn1 levels, and lower H-Fn levels. Functionally, M1 macrophages are less efficient in iron uptake and release and have a more limited response to extracellular iron deficiency or excess than M2 macrophages [224].

The homeostatic iron state of the M1 phenotype is achieved through regulation of the IRE/IRP system. Treatment of murine macrophages with IFNγ and LPS (to promote the M1 phenotype) induces a differential response upon the activities of IRP1 and IRP2 [225]. Treatment with IFNγ/LPS quickly activates IRP1 in an ·NO-dependent manner, and triggers progressive, iron-dependent, IRP2 down regulation [226]. The iron homeostatic response is predominantly mediated by this IRP2 down regulation, since a translational derepression of Fn and an increase in its protein levels are observed; additionally, Tf/TfR1-mediated iron uptake is diminished [227,228,229,230]. The activation of NOX is presumably involved in the decrease in TfR1 expression mediated by LPS [231]. Overall, these studies are consistent with the observation that the microglial M1 phenotype supports iron incorporation through DMT1 and the M2 phenotype via the Tf-TfR1 system [232]. Although it is expected that TfR1 and DMT1 have the same expression pattern, since they both have IREs in the 5′ UTR region of their mRNAs, DMT1 is also stimulated at the transcriptional level by LPS/IFNγ in M1 macrophages, which would explain the antagonistic behavior of DMT1 and TfR1 regulation [233]. Additionally, LPS-treated macrophages reduce Fpn1 expression in an IRP- and ·NO-dependent manner [234]. The rapid IRP1 activation is followed by a decrease, also mediated by ·NO, of c-aconitase/IRP1 mRNA and protein levels [235], configuring the final iron phenotype of M1 macrophages described above.

Additionally, iron modulates differentiation towards one or the other phenotype. Iron overload triggers M1 polarization via an ROS-mediated mechanism [236], increasing TNFα and IL1β secretion [213] and causes M2 macrophages to switch their phenotype to M1 [237]. Accordingly, iron chelation with deferoxamine reduces RNS/ROS release and TNFα and IL1β secretion by microglia [238] and also promotes microglial M2 polarization in APP/PS1 transgenic mice, together with reduced brain iron and Aβ_1–42_ deposition [239]. The brain-permeable iron chelator VK-28 also stimulates microglial polarization towards an M2-like phenotype [240]. Remarkably, the conditional deletion of H-Fn in macrophages reduces LPS/IFNγ-mediated iNOS expression (a marker of M1-polarized macrophages) and increases iron-mediated toxicity [241], suggesting that a higher H-Fn expression in M1 macrophages contributes to the storage and detoxification of exogenously added iron.

As a product of the increase in ·NO production, the M1 phenotype also has quite different metabolic characteristics from the M2 phenotype. Nitric oxide induces disassembly of the [4Fe-4S] cluster on m-aconitase, breaking the flux along the tricarboxylic acid (TCA) cycle, thus triggering a reduction in pyruvate oxidation by pyruvate dehydrogenase and diminishing the protein levels and activity of mitochondrial electron transport chain complexes [242]. Therefore, the M1 phenotype supports energy metabolism based on the oxidation of glucose by glycolysis and the subsequent conversion of pyruvate to lactate. Interestingly, treating microglia with FeCl_3_ upregulates both the expression and activity of 6-phosphofructo-2-kinase/fructose-2,6-biphosphatase 3, which is an enzyme that controls fructose 2,6 bisphosphate levels—the key stimulator of glycolytic flux—suggesting that increased glycolysis and iron retention are interrelated [69].

The iron-induced change to an M1 phenotype may represent an adaptive mechanism that allows microglia/macrophages to survive in a pro-oxidant environment, especially to survive their own ·NO production. The metabolic shift from oxidative phosphorylation to glycolysis decreases the production of ·O_2_^−^ by the mitochondria, which would otherwise react with ·NO to produce the highly toxic peroxynitrite molecule. Higher levels of H-Fn also protect M1 microglia/macrophages from iron-mediated oxidative stress. Supporting this hypothesis, the glycolytic signatures of LPS-treated macrophages are lost under low environmental oxygen tension [243]. Additionally, M2-polarized microglia are more sensitive to ferroptosis, which is an iron- and redox-driven cell death program [244]. M2 microglia have increased levels of 15-lipoxygenase (15-LOX)—an important enzyme for the synthesis of pro-resolving lipid mediators—which also catalyzes the production of an essential pro-ferroptosis lipid signal [245]. Suggestively, M1 resistance to ferroptosis depends on iNOS-mediated ·NO production, and ·NO treatment protects M2 microglia from ferroptosis [244]. The implications of the role of iron in the balance between M1/M2 phenotypes in neurodegenerative diseases remain largely unexplored.

In immortalized microglia, iron potentiates Aβ-induced IL1β secretion (an M1 cytokine) through an ROS- and NFκB-mediated pathway [246]. The Aβ peptide (like LPS and IFNγ) promotes M1 microglial polarization, concomitantly with an increase in NTBI uptake and elevated levels of DMT1 and H-Fn. Additionally, M1 microglia shift their metabolism toward glycolysis, increasing the production of lactate and extracellular acidity, and enhancing pH-dependent iron uptake through DMT1 [232,247]. These glycolytic and iron-retentive microglia are also observed in APP/PS1 transgenic mice [69,247]. Furthermore, in a PD mice model induced by paraquat and maneb, NE depletion by DSP-4 amplifies hippocampal microglial activation and M1 polarization and increases the iron content, correlated with the upregulation of TfR1 and downregulation of Fpn1 [26].

In mice deficient for the NFκB family member c-Rel, which show a late onset parkinsonism preceded by some prodromal PD symptoms, such as intestinal constipation and olfactory impairment [248], an increased expression of M2 microglia/macrophages markers is transiently observed in young, but not older, animals [249]. It remains to be established if this switch from an M2 phenotype to an inflammatory M1 phenotype is a consequence of iron overload caused by phagocytosis of iron-rich, neuromelanin-bearing neurons in the c-Rel KO mice, or of iron-loaded amyloid plaques in the APP/PS1 mice. Overall, the above results indicate that the modulation of M1/M2 polarization is a promising therapeutic alternative for reducing neuroinflammation and dopaminergic neuronal death [250]. Targeting the iron homeostasis regulatory mechanisms could be a feasible alternative.

## 6. A Synergistic Role of Iron Accumulation and Neuroinflammation in Neurodegeneration

Neuroinflammation and brain iron accumulation mutually enhance each other through multiple mechanisms. Beyond translational regulation mediated by the IRE/IRP system, iron transporters can be regulated at the transcriptional or post-translational level by inflammatory mediators. For example, ·NO can S-nitrosylate DMT1 at Cys23 and Cys540, increasing Fe^2+^-uptake activity [251]. Additionally, ·NO also regulates DMT1 protein levels through an indirect mechanism. Parkin, which is an E3 ubiquitin ligase involved in dopaminergic neuron survival, is S-nitrosylated in PD brains and MPTP-injected mice and this modification inhibits its function [252]. Parkin mediates the ubiquitylation of the DMT1B isoform [253,254]. Accordingly, S-nitrosylation of Parkin impairs DMT1 ubiquitination, increasing DMT1 protein levels. Furthermore, treatment with MPP^+^ (the active metabolite of MPTP) results in Parkin S-nitrosylation and elevated DMT1 protein levels in Parkin-expressing human neuroblastoma cells. A similar effect is observed in the SN of MPTP-injected mice [255]. Interestingly, neuronal DMT1 overexpression triggers an increase of Parkin levels in an apparent compensatory response [256].

The expression of DMT1 is transcriptionally enhanced by the transcription factor NFκB [257], whose activation occurs downstream of many cytokine receptors, such as the TNF receptor (TNFR) and the IL1 receptor (IL1R). The activation of NFκB by inflammatory stimuli may play a significant role in iron accumulation by dopaminergic neurons of the SN, which express high levels of TNFR [258]. Interestingly, an increase in the nuclear immunoreactivity of NFκB was observed in PD patients’ brains or in animal models of this disease [259]. Remarkably, treatment with ebselen, which is a selective DMT1 blocker, reduces iron deposition in the SN of LPS-treated mice and prevents neuronal loss and motor deficits [251], suggesting that DMT1-mediated iron entry is relevant in neuroinflammation-mediated neuronal death. Moreover, AD post-mortem tissue displays an increased expression and/or activation of NFκB, particularly in regions preferentially affected in AD [260]. This increased expression correlates with an increased DMT1 expression, both in post-mortem tissue and in transgenic APPsw mice [261].

In PD, a self-perpetuating cycle between neurodegeneration and neuroinflammation is also sustained by neuromelamin (NM) released from dead dopaminergic neurons. Neuromelanin is an insoluble pigment formed by oxidized metabolites of dopamine with a remarkably avidity for Fe^3+^ ions that accumulates with aging, particularly in the SN and LC [262]. Neuromelanin-containing neurons are selectively vulnerable to neurodegeneration [263]. The engulfment of extracellular NM by microglia [264] induces NFκB-dependent microglia activation [265], and triggers mesencephalic neuronal death [266].

The co-occurrence of iron accumulation and neuroinflammation can exacerbate neuronal death. Therefore, the use of iron chelators during neuroinflammation protects the brain from iron overload, reduces microglial activation, and improves cognitive functions in rodents [267,268,269]. As iron catalytically converts H_2_O_2_ and ·O_2_^−^ to the highly toxic hydroxyl radical through the Haber–Weiss reaction, their accumulation could enhance neurotoxicity mediated by glial NOX products.

**Figure 3:**
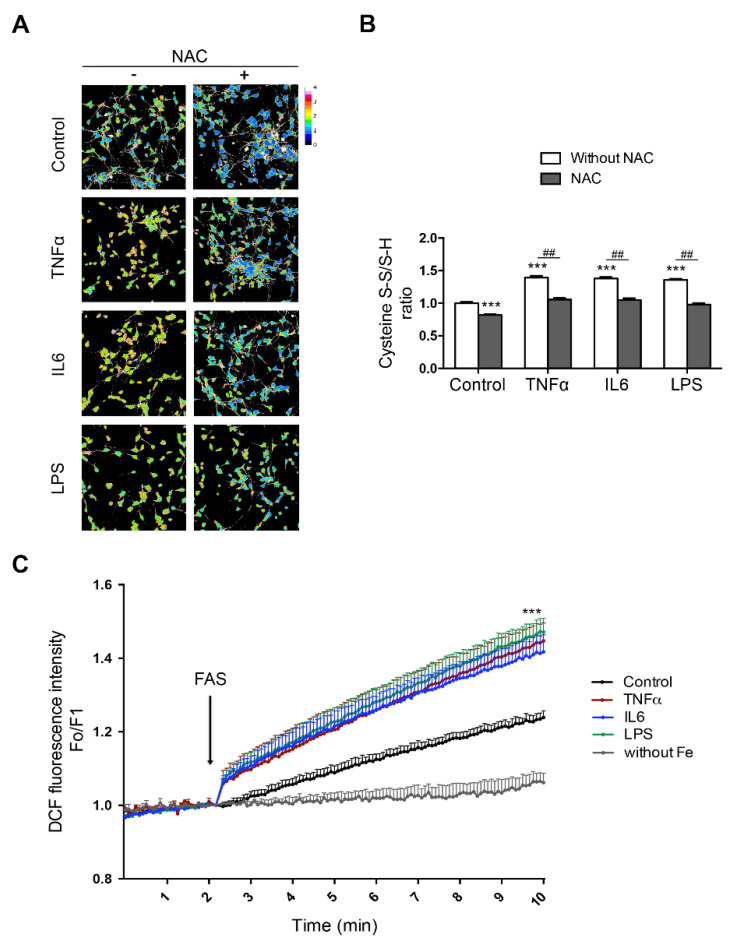
Pro-inflammatory cytokines enhance iron-mediated reactive oxygen species (ROS) production. Hippocampal neurons (7 DIV) were treated with tumor necrosis factor (TNF)α (50 ng/mL), IL6 (50 ng/mL), or lipopolysaccharide (LPS) (1 μg/mL) for 18 h in the presence or absence of 0.5 mM N-acetylcysteine (NAC). (**A**) The oxidative tone was evaluated by the amount of reduced and oxidized cysteine in proteins. Maleimide-Alexa 488 (green) was used to detect reduced cysteines and maleimide-Alexa 568 (red) to detect oxidized cysteines. The ratio between red and green fluorescence was transformed (ImageJ program) into a thermal scale (right hand bar) in which a shift from blue to red to white implies a higher degree of oxidation. (**B**) Quantification of the reduced/oxidized cysteine ratio. (**C**) Increased dichlorofluorescein (DCF) fluorescence, which is a dye sensitive to ROS production, was evaluated after the addition of ferric ammonium sulfate (FAS). Fluorescence data were collected in a microfluorometer plate reader and the ratio between fluorescence (F) and initial fluorescence (F_o_) was plotted. Values represent the mean ± SEM (*n* = 120 neurons, from three independent experiments). *** *p* < 0.001 compared to the control and ## *p* < 0.01 compared with the conditions without or with NAC. For protocol detail see [137].

Iron also promotes microglial activation and NOX2-dependent ·O_2_^−^ production, and in turn, microglia activation contributes to selective iron-mediated neurotoxicity in mixed midbrain-derived primary cultures [270]. NOX2 activation is also involved in paraquat-mediated microglial activation by iron, and microglial cells are essential for enhanced dopaminergic cell death triggered by paraquat/iron treatment [271]. In paraquat- and maneb-treated mice, the NOX inhibitor apocynin restores normal Fpn1 protein levels and inhibits iron accumulation, ameliorating neuroinflammation, lipid peroxidation, and dopaminergic neurodegeneration [272]. Similarly, iron also increases LPS-neurotoxicity when administered to co-cultures of primary neurons and microglia, and neuronal death can be reversed by NOX2 and NOX4 inhibition [273]. Preliminary results from our laboratory also show a synergistic role of neuroinflammation and iron in downstream oxidative stress (Figure 3).

Treatment of hippocampal neurons with pro-inflammatory cytokines (IL6 and TNFα) or with LPS increases the fraction of oxidized cysteines; this increase is abrogated by pretreatment with the antioxidant N-acetylcysteine (NAC, Figure 3A,B). These changes make neurons more prone to oxidative damage, since an increase in their iron content, together with increased ROS production, fosters the production of the highly reactive hydroxyl radical. Accordingly, the detection of hydroxyl radicals with dichlorofluorescein (DCF) indicated that the pre-treatment with IL6, TNFα, or LPS enhanced its production after incubation with iron (Figure 3C). Hence, elevated levels of neuronal iron can act synergistically with cytokine-mediated ROS production to overwhelm antioxidant defenses.

## 7. Conclusions

**Figure 4:**
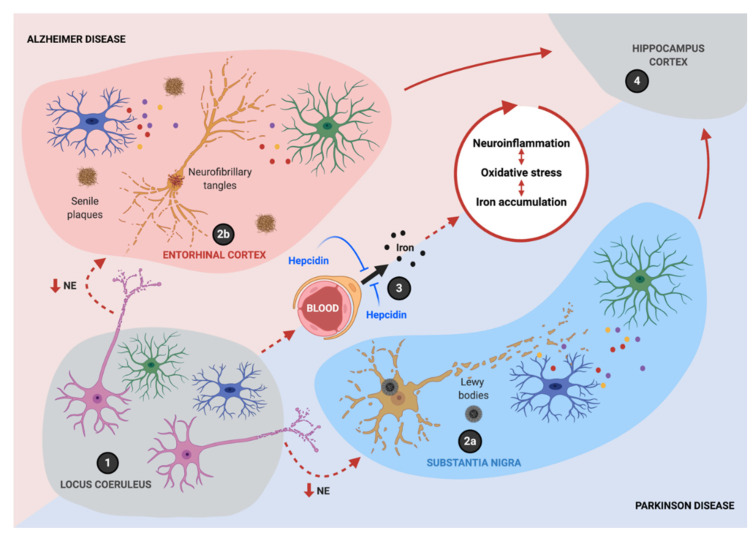
Iron and inflammation are intertwined in a bidirectional relationship during neurodegeneration. The neurodegenerative process starts with the loss of immune homeostatic mechanisms, partially due to decreased norepinephrine (NE) neurotransmission after the degeneration of the locus coeruleus (LC) (1). It continues with neuronal death in intrinsically sensitive areas, such as the substantia nigra (SN) (2a) and the entorhinal cortex (2b), fueled by a positive feedback loop between neuroinflammation, oxidative stress, and iron accumulation (the wheel at the center). As the disease progresses, neurodegeneration continues to other brain regions, such as the hippocampus and the cortex (4). Hepcidin can suppress the main pathologies in experimental Alzheimer’s disease (AD) and Parkinson’s disease (PD) through the inhibition of iron entry at the blood–brain barrier (BBB) (3). Created with BioRender.com.

Connected through an intricate network of molecular interactions, neuroinflammation and iron accumulation establish a noxious circle that sustains the progressive neurodegeneration process observed in AD and PD (Figure 4). Iron promotes the M1 pro-inflammatory phenotype in microglia and macrophages, characterized by the expression of iNOS. Moreover, iNOS-mediated ·NO production is essential for the adaptive remodeling of iron homeostasis and metabolic pathways in M1 microglia/macrophages that concur to ferroptosis resistance. These changes make the endurance of neuroinflammation over time possible, even under oxidative stress conditions that would be toxic to neighboring cells such as neurons. Additionally, ·NO can disrupt the Fe-S cluster in c-aconitase, activating IRP1, even in iron-sufficiency conditions, thus potentiating mitochondrial iron accumulation and oxidative stress, ultimately leading to neuronal death.

Further knowledge on the molecular hierarchy that supports the relationship between neuroinflammation and iron overload will open new therapeutic avenues that allow for the disruption of this circle in AD and PD. Restraining iron entry to the CNS by hepcidin treatment and conservative brain iron chelation are two possible strategies for the treatment of these devastating diseases.
